# Insights Into Non-coding RNAs as Novel Antimicrobial Drugs

**DOI:** 10.3389/fgene.2019.00057

**Published:** 2019-02-22

**Authors:** Gisela Parmeciano Di Noto, María Carolina Molina, Cecilia Quiroga

**Affiliations:** Universidad de Buenos Aires, Consejo Nacional de Investigaciones Científicas y Tecnológicas, Instituto de Investigaciones en Microbiología y Parasitología Médica (IMPAM), Facultad de Medicina, Buenos Aires, Argentina

**Keywords:** sRNA, CRISPR-Cas, antimicrobial, RNA, delivery

## Abstract

Multidrug resistant bacteria are a serious worldwide problem, especially carbapenem-resistant *Enterobacteriaceae* (such as *Klebsiella pneumoniae* and *Escherichia coli*), *Acinetobacter baumannii* and *Pseudomonas aeruginosa*. Since the emergence of extensive and pan-drug resistant bacteria there are few antibiotics left to treat patients, thus novel RNA-based strategies are being considered. Here, we examine the current situation of different non-coding RNAs found in bacteria as well as their function and potential application as antimicrobial agents. Furthermore, we discuss the factors that may contribute in the efficient development of RNA-based drugs, the limitations for their implementation and the use of nanocarriers for delivery.

## Introduction

In the year 2014, the World Health Organization reported the critical problem of antibiotic resistant bacteria (World Health Organization [WHO], 2014). The global resistance levels of bacterial isolates have climbed unrelentingly in the last decades regardless of their source, i.e., clinical settings, in-patients, community, food-related or environmental niches. This led to the increase in the overall morbidity and mortality due to multidrug resistant bacteria (MDR) infections (Baquero et al., 2015; Woolhouse et al., 2016). Throughout the years, misused and abused antimicrobial drugs have led to the selection of resistant strains difficult to eradicate (Baquero et al., 2015). As a result, bacteria have evolved into extensive- (XDR) or pan-drug resistant (PDR) phenotypes.

The Center for Disease Control and Prevention has classified some gram-negative bacteria as urgent or serious threats for public health. Among them, *Enterobacteriaceae* resistant to carbapenems (CRE) or to extended spectrum beta-lactamases (EBSL), multidrug resistant *Acinetobacter* and *Pseudomonas* species present serious hazards. The lack of novel antimicrobial drugs available in the market or the drug development pipeline to combat these pathogens, the high cost of discovering and developing new compounds and the fast evolution of bacterial population to resistant phenotypes are particularly worrisome. Therefore, novel approaches to battle these pathogens are currently encouraged (World Health Organization [WHO], 2014). One promising strategy is the use of RNA-based therapies. This review examines the current situation of non-coding RNA (ncRNA) elements as antimicrobial agents and discusses some strategies and limitations for their implementation.

## Non-Coding RNAs as Therapeutics Agents

**FIGURE 1:**
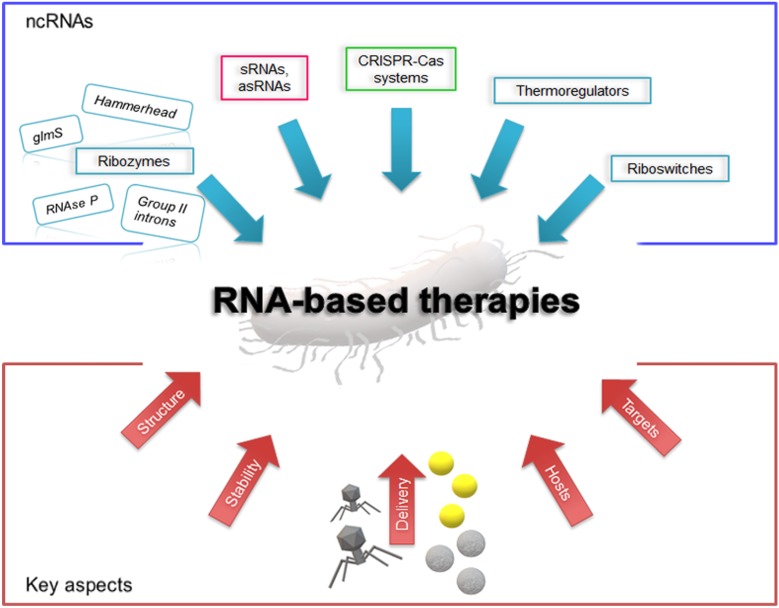
Schematic representation of the current scenario of RNA-based therapies. The blue box shows the outline of candidate antimicrobial ncRNA elements; in the red box are illustrated some key aspects to be considered for RNA drug design and development. Yellow spheres depict gold nanoparticles and gray spheres represent polymeric nanoparticles.

Since a few decades ago, RNA molecules have been foreseen as potential drugs against pathogens. With the characterization of novel ncRNAs in bacteria, this strategy seems more plausible. Among the ncRNA molecules studied for their therapeutic potential are the ribozymes hammerhead, group II introns, glmS, and RNAse P (Figure 1) (Cui and Davis, 2007; Ferré-D’Amaré, 2010; Lambowitz and Zimmerly, 2011; Hammann et al., 2012; Altman, 2014; Khan et al., 2016). One of the most studied ribozymes is RNAse P. Its activity and interaction with external guide sequence as therapeutics against MDR bacteria has been extensively reviewed elsewhere, and interesting advances in the field have been reported (Forster and Altman, 1990; Kirsebom and Svärd, 1992; Svärd and Kirsebom, 1993; Altman, 2014; Davies-Sala et al., 2015). The approach for the use of this ribozyme is based on the delivery of nuclease-resistant analogs, such as locked nucleic acids/DNA co-oligomers or phosphorodiamidate morpholino oligonucleotide EGSs conjugated to permeabilizer peptide (PPMO), that induce a RNAse P-mediated degradation of the target mRNA once introduced in the host. Further advances using this strategy will most likely provide interesting results that will contribute in developing novel RNA-based drugs.

Hammerhead ribozymes have been used to develop antiviral compounds; however, their use against bacteria has not been considered yet (Hammann et al., 2012). Group II introns are self-splicing elements that in the presence of its cofactor can retrotranspose to novel target sites within a genome (Lambowitz and Zimmerly, 2011). Several attempts were made to use these ribozymes as vehicles for the delivery of cargo genes to inhibit cell growth or promote cell death (Plante and Cousineau, 2006; Mohr et al., 2013). One particular subclass of group II introns, C-attC, has the peculiar ability to insert downstream of DNA secondary structures adjacent to antimicrobial gene cassettes located in integron platforms (Centrón and Roy, 2002; Quiroga et al., 2008). The ability exhibited by C-attC group II introns to selectively insert within gene cassettes suggests that they could be employed as vectors to deliver genetic material at specific target sites. Last, the glmS ribozyme has also been a subject of study as an antimicrobial drug. It has been reported that in the presence of carba-α-D-glucosamine it can promote mRNA degradation and inhibit cell growth (Ferré-D’Amaré, 2010; Schüller et al., 2017). Although all these RNA elements have promising features that could be adapted to engineer RNA based drugs, further advances in their delivery are necessary.

The recent upsurge of other functional ncRNAs in bacteria have revealed their essential role in the regulation of different processes, such as cell physiology, defense, horizontal gene transfer, virulence, etc (Gottesman and Storz, 2011; Storz et al., 2011; Caldelari et al., 2013; Fröhlich and Papenfort, 2016). Since many ncRNAs are key regulatory elements, they are currently considered for designing novel therapeutic strategies. These RNAs are commonly small in size (<500 nt), and can either act in *cis* of the target messenger RNA (thermoregulators, riboswitches) or in *trans* [small RNAs, antisense RNAs, clustered regularly interspaced short palindromic repeats (CRISPRs)] (Figure 1). Riboswitches and thermoregulators control the expression of an adjacent mRNA upon sensing physical or chemical signals (Winkler et al., 2002; Chowdhury et al., 2006). The environmental effect or the presence of specific molecules lead to structural modifications in the 5′-UTR of a target mRNA that can either release or sequester the ribosome binding site, resulting in the activation or repression of translation. While thermoregulators are mostly temperature-sensitive RNAs that respond to heat or cold shock, riboswitches are more complex elements that regulate a wide variety of genes. Some riboswitches, such as the guanine riboswitch, have shown promising results as targets for novel antimicrobial compounds against the pathogen *Clostridioides difficile* (Yan et al., 2018). Also, it can regulate the expression of aminoglycoside antibiotic-resistance genes (Jia et al., 2013; Rekand and Brenk, 2017). Mechanistic insight into these RNA sensors and their use as antimicrobials can be found in comprehensive reviews (Chowdhury et al., 2006; Rekand and Brenk, 2017). Two additional ncRNA elements, sRNA and CRISPRs, have lately drawn more attention as potential RNA-based antimicrobial drugs. In the following sections, we will focus on their use, strength and limitations.

## Small Non-Coding RNAs in Bacteria

Small non-coding RNAs (sRNA) are short RNAs that regulate post-transcriptionally gene expression. These RNAs can be encoded in the opposite strand of the target mRNA (known as antisense or *cis* sRNA), or encoded in *trans* to the target mRNA. The *trans* acting sRNA, or simply sRNAs, are RNA regulators frequently found in bacteria that interact by imperfect base pairing with its target mRNA. Their regulation process usually involves the chaperon protein Hfq, as well as ProQ and CsrA (Wagner and Romby, 2015; Olejniczak and Storz, 2017), albeit interactions with other chaperons and *cis* sRNAs have also been reported (Opdyke et al., 2004; Ross et al., 2013; Ellis et al., 2015). These proteins participate in the sRNA and its target mRNA interaction, in mRNA translation or during RNA decay. As a result, sRNAs can repress translation by binding to the initiation target site, by sequestration of the ribosome standby site, or by facilitating mRNA degradation with ribonucleases; they can also activate translation by exposing a sequestered ribosome binding site or protecting a mRNA by masking a ribonuclease cleavage site (Gottesman and Storz, 2011; Storz et al., 2011; Caldelari et al., 2013).

Several studies have shown that sRNAs regulate a wide variety of genes that code for proteins involved in processes related to physiology, metabolism, stress responses or quorum sensing (reviewed in Gottesman and Storz, 2011; Storz et al., 2011; Caldelari et al., 2013; Fröhlich and Papenfort, 2016). Many of them are capable of regulating more than one target mRNA, which unveils a complex sRNA-based network (Storz et al., 2011). Furthermore, recent studies have suggested that approximately half of the mRNAs are regulated by sRNAs (Hör and Vogel, 2017), which showcase their important role in post-transcriptional control. sRNAs regulators provide different benefits to the host, such as reduced metabolic cost and a tighter and faster gene regulation, that help bacteria to adapt to new environments (Beisel and Storz, 2010). Thus, sRNA-mediated regulation is currently regarded as RNA-based drug targets. In this regard, Na et al. (2013) designed several synthetic sRNAs targeting various mRNAs RBS, which modulate gene expression in different *Escherichia coli* strains. Since then, several studies on the application of sRNAs in metabolic engineering and synthetic biology have been published (reviewed in Villa et al., 2019).

Other appealing target candidates include virulence and resistance genes as well as mobile elements, thus they have become appealing candidates. In this regard, it has been reported that some sRNAs are involved in antibiotic uptake (GcvB, RyhB, MicF, ErsA), drug efflux (DsrA RydC, SdsR, NrrF), biofilm formation (RprA OmrA/B, McaS, RybB, RydC), and modification of lipopolysaccharide and cell wall synthesis (MgrR, MicA, Sr006). While most of these sRNAs have been extensively studied in *E. coli* and *Salmonella* strains (reviewed in Dersch et al., 2017), there is scarce information about their activity in other bacteria. The identification of sRNAs related to antimicrobial resistance genes and their mechanisms of dissemination exposes a new strategy for the delivery of synthetic sRNAs to XDR and PDR bacteria.

## The CRISPR-Cas Systems in Bacteria

CRISPR-Cas systems are part of the immune system of bacteria and provide protection against mobile genetic elements. Its immunity is based on the specific sequence recognition of foreign DNA or RNA by base pairing with short guide RNAs (32–35 nt), followed by the cleavage of the target sequence by CRISPR-associated protein (encoded by the *cas* genes). There are two classes and several types of CRISPR-Cas systems, which are usually composed of a *cas* operon adjacent to a CRISPR array (Koonin et al., 2017). Such array consists of direct repeats interspaced by the DNA invader-derived guide sequences that anneals with the exogenous material (Jackson et al., 2017; Hille et al., 2018 and references within). In recent years, the CRISPR-Cas machinery has been repurposed for gene editing and interference. These systems have a highly sequence-specific targeting ability that inspired the research community to use them as novel antimicrobial agents. The unique activity of CRISPR-Cas systems regards them as elements that can either attack resistance genes or populations of unwanted pathogenic bacteria, while preventing the eradication of bacteria that might be beneficial (Bikard and Barrangou, 2017; Goren et al., 2017; Greene, 2018).

To date, a few CRISPR guide RNAs have been designed to target virulence factors, antimicrobials determinants or essential chromosomal genes from specific pathogens, such as *E. coli* or *Staphylococcus aureus* (Bikard et al., 2014; Citorik et al., 2014; Gomaa et al., 2014). These systems were employed to efficiently target a particular DNA sequence resulting in the introduction of chromosome deletions in different pathogens, which consequently led to cell death or to the reduction in the population of unwanted bacteria (Vercoe et al., 2013; Bikard et al., 2014; Citorik et al., 2014; Gomaa et al., 2014; Hampton et al., 2016). Vercoe et al. (2013) observed that a guide or CRISPR RNA (crRNA) programmed to target a large horizontally acquired island in *Pectobacterium atrosepticum* activated the endogenous CRISPR-Cas system and promoted the loss of both islands and the accessory genes encoded within. Moreover, double-stranded DNA breaks caused by the Cas machinery made CRISPR-Cas target the bacterial chromosome and resulted in the inhibition of cell growth and a filamentation phenotype (Vercoe et al., 2013). Although it has been confirmed that resistance genes can be eliminated using this technique (Bikard et al., 2014; Citorik et al., 2014), spontaneous point mutations in bacterial genomes might affect the action of synthetic guide CRISPR RNAs or endogenous CRISPR-Cas systems. Therefore measures to counteract these effects during new drug development should be contemplated.

## Considerations on the Design of RNA-Based Antimicrobial Strategies

The development of RNA-based antimicrobial strategies requires the understanding of the factors involved in the mechanisms and activities of each RNA element, the determination of their specificity to ascertain that no off-targets and unexpected events occur, and the evaluation of the impact that introducing these RNAs may cause to the host. Most studies have been limited to reference strains, such as *E. coli* MG1655, whereas only few of them have been done using clinical isolates (Bikard et al., 2014; Citorik et al., 2014; Gomaa et al., 2014; Chan et al., 2017; Dersch et al., 2017). The extensive genome sequencing projects in antimicrobial resistant pathogens revealed that clinical isolates have large, versatile and plastic genomes that encode an assortment of cellular factors. The process of selecting a target mRNA and designing RNA-based drugs, either using sRNAs or CRISPR guide RNAs, will most likely require a subsequent validation in different bacteria (Figure 1).

A special consideration should be placed on the selection of the target mRNAs (Figure 1). Most mRNAs are good candidates for RNA-based antimicrobials; however, current approaches for developing drugs are aiming for specific targets that have little or no effect on the host microbiota (Langdon et al., 2016; Lichtman et al., 2016). To overcome this problem, a safe approach involves directing the attack to specific genes that will only have an impact on pathogenic bacteria. Therefore, virulence genes, antimicrobial resistant determinants, mobile genetic elements or genes involved in horizontal transfer are ideal candidates. Designing sRNAs or guide RNAs that hybridize specifically with those genes will limit the effect on microbial flora even if they are introduced in other host cells.

Furthermore, the design of synthetic RNAs should take into consideration their stability in the cell, as well as their folding into proper structures (Figure 1). Previous studies have shown that single strand RNAs are more stable when their extremities are protected by stem-loop structures, which improves their survival in the cell (Majdalani et al., 1998). Although this increases their stability, they are not exempted of the effects of the host degradation machinery. In this regard, RNAs that bind to specific proteins (e.g., Hfq or Cas) can be protected from the action of RNAses, which will increase RNA survival in the cell and the execution of the desired tasks. Therefore, functional and structural studies on Hfq interaction with synthetic sRNAs or between guide RNAs and Cas proteins will help to optimize their activity and reduce undesired degradation.

Despite the fact that chaperons and cofactors can provide stability to the candidate RNAs, delivery of RNPs may prove difficult in bacterial cells. Alternatively, some studies have suggested the use of endogenous CRISPR-Cas systems against XDR and PDR bacteria. A caveat in this strategy is that CRISPR-Cas systems are not conserved in bacterial species (Koonin et al., 2017) and previous confirmation of their presence in the host will be necessary.

## RNA Delivery in Bacteria

The need to explore new delivery systems capable of overcoming the challenges of specificity, selectivity for targeting and efficiency has appeared. Transport of genetic material from an extracellular environment into cytosolic compartment is a complex task specially when referred to transport across bacteria barriers, outer membrane (in gram-negative bacteria), the cell wall and the cytoplasmic membrane (Chen and Dubnau, 2004). Synthetic nanocarriers and bioinspired vehicles, such as bacteriophages, have been investigated for their use in drug and gene delivery systems (Figure 1). Bacteriophages are viruses with a highly efficient ability for compressing and wrapping DNA to form compact particles of 28 nm (MS2), 200 nm (T4) or 890 nm (M13) (Karimi et al., 2016). Based on the potential of these viruses to naturally act as carriers, they have been employed in the transfer of genetic information. Phage therapy has been revisited as an alternative to antibiotics for treating bacterial infections in different models as well as implemented in phase I and II of clinical trials (reviewed in Lin et al., 2017). Non-lytic bacterial cellular death was reported employing phagemid constructs that can carry different antimicrobial compounds and target specific bacteria (Krom et al., 2015). The authors showed that this approach led to a significant reduction in bacterial cell viability *in vitro* and an 80% survival rate in a murine peritonitis infection model, which are promising results.

Toward ncRNA-based antimicrobial therapeutics, Na et al. (2013) showed that custom sRNA cassettes carrying the antisense sequence of a target mRNA and an Hfq-binding motif it is possible to modulate gene expression in different *E. coli* strains. Based on these findings Bernheim et al. (2016) developed a protocol for synthetic sRNA delivery in *E. coli* cells using a phagemid construct and a non-lytic M13 phage that upon encapsulation can infect a population.

On the other hand, three research groups have assessed the delivery of CRISPR-Cas system using phage particles as vectors that seizes the specificity of phages for their hosts (Bikard et al., 2014; Citorik et al., 2014; Yosef et al., 2015). Citorik et al. (2014) used CRISPR-Cas technology and created RNA-guided nucleases targeting antibiotic resistance and virulence determinants in carbapenem-resistant *Enterobacteriaceae* and enterohemorragic *E. coli.* This strategy involved the delivery of RNA-guided nucleases using a bacteriophage or a conjugative plasmid. Bikard et al. (2014) used a phage-encoded CRISPR-Cas9 to target antibiotic resistance genes in strains of *Staphylococcus aureus*. Both groups confirmed their results with *in vivo* experiments, in a *Galleria mellonella* infection model and a mouse skin colonization model (Bikard et al., 2014; Citorik et al., 2014). Lastly, Yosef et al. (2015) improved the delivery model by combining the use of a λ prophage and the lytic phage T7. They used *E. coli* as a host and delivered the CRISPR cascade genes and *cas3* of a type I-E CRISPR-Cas system along with the guide crRNAs designed to target the beta-lactam resistance genes *bla_NDM_*_-1_ and *bla*_CTX-M-15_. They proposed to sensitize *E. coli* cells to β-lactam antibiotics while simultaneously conferring a selective advantage to sensitized bacteria by protecting them from lytic phages with an engineered CRISPR-Cas system delivered by a λ prophage. Therefore, when *E. coli* cells were infected with a T7 phage, only bacteria that were sensitized and had an active CRISPR-Cas system were able to resist the infection. The authors stated that the use of this technology would reduce multi-drug resistant populations, overcome the resistance problem and re-purpose several antibiotics that are no longer used. However, some limitations regarding conjugation efficiency, host range and phage resistance suggest that new delivery vehicles need to be tested. In this regard, nanotechnology offers promising options of nanocarriers that should be explored for antimicrobial delivery systems, a wide variety of materials, and the possibility to improve targeting designed to specifically reach bacterial cells. Of note, extracellular vesicles (EVs) derived from phage-sensitive bacteria have also been proposed as potential extra opportunities in phage therapy. EVs can be administered prior to the phages to enhance the targeting of bacteria and even enable the infection of novel bacterial host targets (Liu et al., 2018).

Non-viral nanoparticles have been tested as nanocarriers to achieve the incorporation of genetic material in bacteria. For instance, encapsulation of plasmid DNA with different molecular weights of chitosan (chitosan-pDNA NPs) resulted in different NP sizes (457 to 820 nm) that greatly enhanced transformation efficiency in *E. coli* cells compared to naked DNA (Bozkir and Saka, 2004). Further showing the potentiality of nanoparticles and chitosan to introduce genetic material in bacterial cells, other research groups have evaluated the efficiency of plasmid DNA delivery using electrospray of chitosan-pDNA NPs into non-competent vs. competent *E. coli* (Abyadeh et al., 2017), electrospray of gold NPs (GNPs) in non-competent *E. coli* (Lee et al., 2011), and transformation of GNPs – pDNA conjugates by high temperature and friction forces of the Yoshida effect in gram positive and gram negative bacteria (Kumari et al., 2017). However, to the best of our knowledge they have not been tested yet using ncRNAs as cargo.

Although the progress in the field is promising, there are still many questions to be answered. For instance, which nanoparticle will efficiently deliver sRNAs without compromising its activity? How functional and adaptable has to be a synthetic system in order to battle the evolution of bacteria toward antimicrobial resistance?

And in the particular case of CRISPR-Cas systems, is it suitable to use the endogenous machinery of pathogens and deliver only CRISPR RNAs, or is it better to deliver the entire CRISPR-Cas machinery? Which type of CRISPR-Cas is more efficient? How efficient is the delivery of these systems with bacteriophages? In this regard, it is well-known that bacteria can resist phage infections using other strategies besides CRISPR-Cas, i.e., by spontaneous mutations of sensitive cells independently of the action of the virus, with restriction and modification systems, masking of membrane receptors or with toxin/antitoxin systems. Moreover, recent reports have revealed that bacteria can encode anti-CRISPR proteins in prophages, which could affect the efficiency of the CRISPR-Cas system (Labrie et al., 2010; Seed, 2015; van Houte et al., 2016; Borges et al., 2017; Oechslin, 2018; Pawluk et al., 2018). There are no studies yet on how these mechanisms would work in face of these therapies.

## Concluding Remarks

The antimicrobial resistance problem is a crucial global issue that needs to be addressed. The development of alternative strategies to battle bacterial pathogens are of outmost importance. RNA-based therapies, such as synthetic sRNAs or CRISPR guide RNAs, are attractive strategies to tackle this problem. Both approaches can target accessory genome of pathogenic bacteria, in particular extended spectrum beta-lactams, carbapenems or colistin resistance genes. However, it is important to develop systems that not only are successful for delivering highly effective RNA elements but that can also be rapidly modified upon bacterial acquisition of novel resistances and limits the selection of MDR bacteria. Furthermore, a combined system targeting several mRNAs in a coordinate manner would ideally be more robust. In this regard, the CRISPR-Cas systems have revolutionized the world of microbiology, and their use in the fight against antibiotic multiresistance is going to be without a doubt a powerful tool. Notwithstanding, more studies are indeed necessary to be able to deliver these RNAs with high specificity and achieve a clinically relevant efficacy. The advances on the activity of sRNA and CRISPR-Cas systems have raised the issue of their use as antimicrobial drugs, further progress in the RNA and nanotechnology field are necessary to answer all these questions.

## Author Contributions

CQ, GPDN, and MCM wrote the manuscript. All the authors discussed the content, contributed to manuscript revision, and read and approved the submitted version.

## Conflict of Interest Statement

The authors declare that the research was conducted in the absence of any commercial or financial relationships that could be construed as a potential conflict of interest.
